# Multiparametric magnetic resonance imaging for radiotherapy response evaluation in high-risk soft tissue sarcoma: A pilot study

**DOI:** 10.1016/j.phro.2025.100818

**Published:** 2025-07-25

**Authors:** Milan van Meekeren, Petra J. van Houdt, Marta Fiocco, Jessica M. Winfield, Christina Messiou, Birthe C. Heeres, Hans Gelderblom, Neeltje Steeghs, Rick Haas, Kirsten van Langevelde

**Affiliations:** a Department of Medical Oncology, Leiden University Medical Center, Leiden, the Netherlands; b Department of Radiation Oncology, The Netherlands Cancer Institute, Amsterdam, the Netherlands; c Mathematical Institue, Leiden University, Leiden, the Netherlands; d Department of Biomedical Data Science, Section Medical Statistics, Leiden University Medical Center, Leiden, the Netherlands; e Princess Máxima Center for Paediatric Oncology, Utrecht, the Netherlands; f MRI Unit, Royal Marsden NHS Foundation Trust, Surrey, United Kingdom; g Division of Radiotherapy and Imaging, The Institute of Cancer Research, London, United Kingdom; h Department of Radiology, The Netherlands Cancer Institute, Amsterdam, the Netherlands; i Department of Medical Oncology, The Netherlands Cancer Institute, Amsterdam, the Netherlands; j Department of Medical Oncology, University Medical Center Utrecht, Utrecht, the Netherlands; k Department of Radiotherapy, Leiden University Medical Center, Leiden, the Netherlands; l Department of Radiology, Leiden University Medical Center, Leiden, the Netherlands

**Keywords:** Soft tissue sarcoma, Radiotherapy, Magnetic resonance imaging, Treatment response evaluation, Repeatability

## Abstract

**Background and purpose:**

Soft tissue sarcomas (STS) are rare mesenchymal tumors for which no clinically validated quantitative magnetic resonance imaging (qMRI) parameters exist yet.

This study explores repeatability and association with histopathology of qMRI parameters during and after neo-adjuvant angiogenesis inhibition (oral pazopanib) and radiotherapy for localized, high-risk STS.

**Materials and methods:**

For fifteen patients, qMRI parameters, including apparent diffusion coefficient (ADC), volume transfer constant (K^trans^) and T_2_ relaxation times were acquired twice at baseline (B1 and B2), twice during neo-adjuvant treatment and pre-surgery. For all three parameters, the mean was determined per tumor. Subsequently, repeatability coefficient (RC or %RC) was assessed from B1 and B2 mean values. Mixed models were estimated to study the association between percentage viable cells from histopathology and absolute change from baseline (ΔqMRI) for ADC mean and percentage change from baseline (%ΔqMRI) for T_2_ and K^trans^ at each time point.

**Results:**

RC was 0.17 × 10^-3^ mm^2^/s for ADC and %RC was, 5 % and 65 % for T_2_ and K^trans^, respectively.

The changes in mean ADC and T_2_ showed both increases and decreases at each timepoint, whereas mean K^trans^ predominantly showed decreases. ΔqMRI for ADC mean, %ΔqMRI for T_2_ mean and %ΔqMRI for K^trans^ mean showed no statistically significant association with % viable cells.

**Conclusion:**

This pilot study reported relatively low repeatability coefficients for ADC and T_2_ and a higher repeatability coefficient for K^trans^ and showed heterogeneous changes in qMRI parameters in fifteen STS patients, however with no association between these parameters and percentage viable cells.

## 1 Introduction

Soft tissue sarcomas (STS) are rare malignant tumors of mesenchymal origin. The multitude of clinical STS trials and expanding neo-adjuvant treatment arsenal call for accurate and ideally minimally invasive methods of assessing treatment response, preferably early during the treatment course. In this regard, quantitative magnetic resonance imaging parameters (qMRIs) [1] derived from functional MRI techniques could be potential biomarkers.

Functional MRI techniques like diffusion-weighted imaging (DWI) and dynamic contrast-enhanced (DCE) imaging are used to characterize underlying biological properties of tumors and their micro-environment. From DWI, the apparent diffusion coefficient (ADC) can be calculated, which is related to the cellularity of the tumor [2]. From DCE imaging, quantitative pharmacokinetic parameters can be derived, such as K^trans^, which is the volume transfer constant between blood plasma and the extravascular extracellular space [3]. Quantitative T_2_ relaxation times, i.e. T_2_ mapping, has the potential to discriminate between different tissue compositions [4]. Previous studies showed changes in T_2_ during treatment for prostate cancer [5,6], and in STS [7]. Both DWI and DCE parameters have been shown to correlate with histopathology when measured post-treatment [8] or as single measurements during treatment [[9], [10], [11]] in STS. However, data with multiple measurements and multiple qMRI parameters during and after treatment still have limited availability.

When using qMRI parameters to measure changes over time, it is important to understand the repeatability of the measurements such that changes due to treatment can be distinguished from measurement errors. Repeatability is assessed with test–retest studies (i.e. two baseline measurements) to define the thresholds of true change versus measurement error [12]. Repeatability data for qMRI parameters in STS is scarce and therefore more data is needed to define thresholds in this rare cancer [13].

The aims of this pilot study were to assess the repeatability of three qMRI parameters (ADC, T_2_ and K^trans^), and evaluate changes in these parameters at multiple time points, in STS patients treated with a neo-adjuvant angiogenesis inhibitor and radiotherapy. A secondary aim was to investigate the association of these parameters with histopathological outcome.

## 2 Materials and methods

### 2.1 Study population

MRI data from patients enrolled in the PASART-2 (NCT02575066) clinical trial, who were treated at the Netherlands Cancer Institute (N = 15), was used [14]. The study protocol and all amendments were approved by the local institutional review board and the study was conducted in accordance with the declaration of Helsinki. Written informed consent was obtained from all patients.

**Fig. 1:**
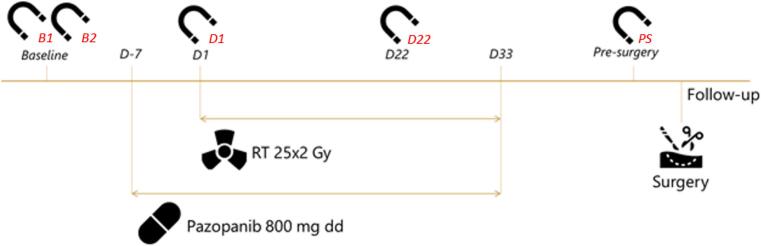
Overview of study procedures and MRI scanning sessions of PASART-2. Abbreviations: B1 = baseline 1, B2 = baseline 2, D-7 = day −7, D1 = day 1, D22 = day 22, D33 = day 33, PS = pre-surgery, RT = radiotherapy, Gy = gray, mg = milligram, dd = once daily. In red, the abbreviations for the 5 MR examinations that will be used throughout this article are given. (For interpretation of the references to color in this figure legend, the reader is referred to the web version of this article.)

Patients in the PASART-2 trial were diagnosed with a localized, high-risk STS and treated with neo-adjuvant radiotherapy (50 Gy in 25 fractions), from day 1 until day 33 (Fig. 1). One week prior to starting radiotherapy, at day −7 (D-7), pazopanib 800 mg daily was started and this was continued until radiotherapy completion (day 33 (D33)). 4–8 weeks later, surgery was performed with the aim of complete tumor resection.

### 2.2 Histopathological examination

Histopathological examination on the resection specimen was performed according to the European Organisation for Research and Treatment of Cancer (EORTC) pathology guidelines [15]. An experienced, EORTC reference STS pathologist noted the percentages of viable tumor cells, hyalinization/fibrosis and necrosis of representative tumor slices. As gold standard or “ground truth” the percentage of viable tumour cells was used. Histopathological response in this study was defined as ≤ 5 % viable tumor cells [16].

### 2.3 MR protocol

Each patient was scanned in 5 sessions on 5 different days (Fig. 1), on a 3 T Philips Ingenia (Philips Healthcare, the Netherlands).

The MR protocol was the same for all examinations and consisted of clinical anatomical sequences as well as quantitative MRI sequences, DWI, T_2_ mapping and DCE imaging. The anatomical sequences were used for tumor delineations and included a sagittal and transverse T_2_-weighted, transverse T*_1_*-weighted, and post-contrast T*_1_*-weighted scan with fat suppression. Details of the scan parameters of the quantitative MRI scans can be found in Supplementary Table 1. Further details about the MR protocol can be found in Supplementary Table 2.

### 2.4 Image analysis

An ADC map was calculated from the DWI data using b-values 600 and 900 s/mm^2^ to avoid including perfusion effects in ADC calculation [17]. A T_2_ map was generated from the multi-echo spin echo data with the scanner software [18]. For quantitative analysis of the DCE data, signal intensities were first converted to gadolinium concentration values using the baseline T*_1_* map [19]. Next, K^trans^ was estimated with the extended Tofts model using the implementation of Murase [20] and a population arterial input function as input [21].

The data were further processed with in-house written software in python. First, the post-contrast T*_1_*-weighted images of all scan sessions were registered to the post-contrast T*_1_*-weighted scan of timepoint *B1* using rigid registration. The rigid registration was based on correlation ratio and applied for a region around the tumor including landmark structures such as nearby bones. The obtained transformations were applied to the ADC map, T_2_ map and K^trans^ map of the same MR examination. For each patient it was visually checked whether an additional registration was needed between ADC, T_2_ or the K^trans^ map. If this was the case, for ADC, the b = 0 s/mm^2^ was first registered to the post-contrast T1-weighted scan. This registration was copied to the ADC map. Similarly, for T_2_ mapping one of the individual echo images was used and for K^trans^ the last dynamic of the DCE series.

For each patient, an initial volume-of-interest (VOI) was defined as the gross tumor volume (GTV) on the radiotherapy planning CT and propagated to the post-contrast T*_1_*-weighted scan at *B1*. This VOI was adjusted to encompass the whole tumor on MRI under supervision of an experienced musculoskeletal radiologist. The *B1* VOI was propagated to the post-contrast T*_1_*-weighted scans of the other scan sessions and adjusted when needed to correct for changes in anatomy of the tumor. In order to analyze the same part of the tumor in all five scan sessions, the delineations were cropped to include only those slices that were available in all scan sessions. For K^trans^, non-enhancing voxels were included in the analysis, to describe the behavior of the lesions as a whole.

### 2.5 Statistical analysis

Histograms were plotted for each qMRI parameter (ADC, T_2_, and K^trans^), showing the distribution of qMRI voxel values within the VOIs of the tumor and compared between scan sessions. The mean of all three qMRI parameters was estimated.

The first two baseline scans were used to estimate repeatability metrics of the mean values of each parameter according to the QIBA guidelines [12]. The RC or %RC were subsequently used as a threshold to determine which changes in qMRI parameters are related to treatment effects. To determine whether absolute or relative changes were appropriate, we determined per qMRI parameter whether the variation in B1 and B2 was dependent on the mean, by calculating the Pearson correlation coefficient between the standard deviation of the differences versus the mean values [22]. If the correlation was significant, the %RC was used as a threshold, otherwise RC [12].

The absolute change from baseline (ΔqMRI) was obtained for each qMRI parameter at each time point by subtracting the B1 values from later qMRI parameter values:

$$\Delta \text{qMRI}={\text{qMRI}}_{t}-{\text{qMRI}}_{\text{B1}}$$

Similarly, the relative change from baseline (%ΔqMRI) was obtained for each qMRI parameter at each time point by dividing the difference between later qMRI parameter and B1 values with the values at B1:

$$\%\Delta \text{qMRI}=\frac{{\text{qMRI}}_{t}-{\text{qMRI}}_{\text{B1}}}{{\text{qMRI}}_{\text{B1}}}∙100$$

For patients without a B1 scan (N = 2), B2 values were used in this equation instead.

To account for the presence of repeated measures, linear mixed models for the outcomes ΔqMRI of mean ADC, %ΔqMRI of mean K^trans^ and %ΔqMRI of mean T_2_ were estimated. Individual-specific random intercept to account for differences across individuals have been included in each model with different histological predictors as fixed effect: percentage viable cells, percentage necrosis, percentage fibrosis/hyalinization. Furthermore, time was also a fixed effect with each later scan session (D1, D22, PS) being a standard time step away from each other of 1. A total of 9 models with three qMRI outcomes and 2 predictors as fixed effect in each model and patients as random effects have been estimated. For each predictor estimated regression coefficient along with the standard errors are reported. Scatter plots of ΔqMRI of mean ADC or %ΔqMRI of mean K^trans^ or %ΔqMRI of mean T_2_ and percentage viable cells or percentage necrosis or percentage fibrosis/hyalinization were provided. Mixed models have been estimated with the library lme4 [23] and the library lmerTest [24] in R software environment [25]. SPSS version 29 has been used for the remaining statistical analyses.

## 3 Results

### 3.1 Patient characteristics

**Fig. 2:**
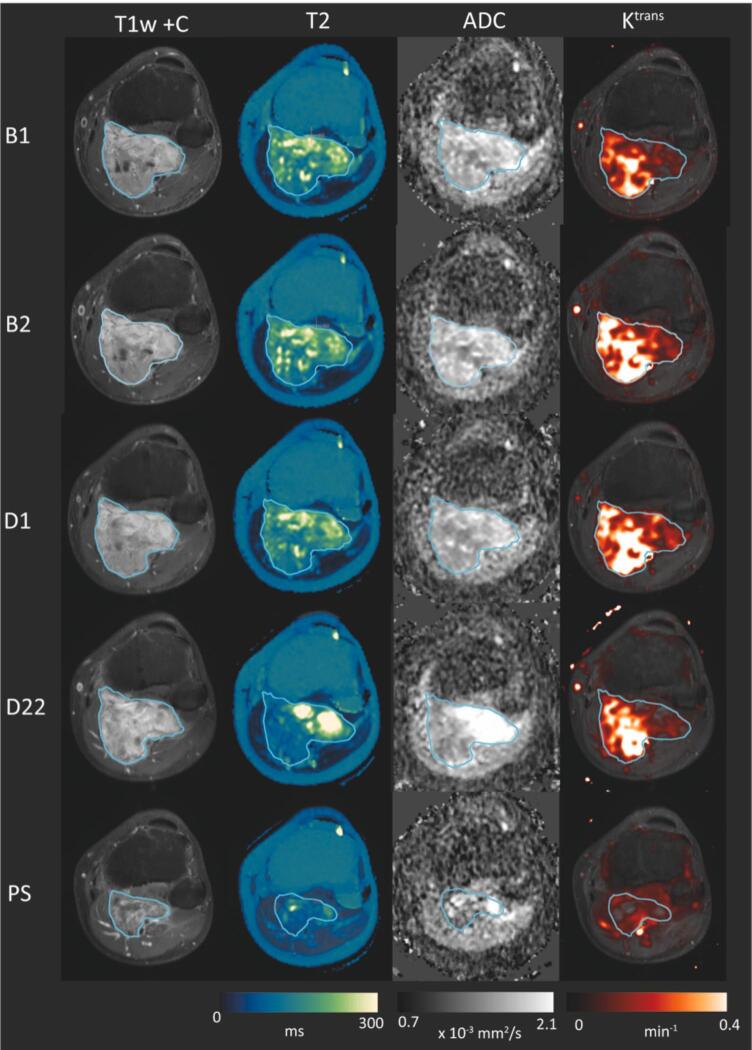
Example images (T_1_w + c, T_2_, ADC and K^trans^) for patient ID13 (responder) are shown for all time points. The tumor is delineated in cyan.

Baseline characteristics and histo-pathological results are described in Table 1. Fig. 2 shows a representative slice of the post-contrast T*_1_*-weighted fat suppressed MRI and three qMRI parameters of ID 13 at all timepoints, T2 maps are displayed with the navia colormap based on consensus recommendations [33]. ADC maps are displayed in a greyscale, whereas Ktrans maps are overlayed on the T1w + C with a red to yellow color map. (For interpretation of the references to color in this figure legend, the reader is referred to the web version of this article.)

**Table 1:** Baseline characteristics and histo-pathological results.

		Baseline characteristics				Histo-pathology – resection specimen			
Case	Sex	Age (years)	Subtype	Location	Largest tumor diameter (cm)*	%VC	%N	%H/F	Slices analyzed
1	Female	49	Myxofibrosarcoma	Lower leg	3.9	60	35	5	8
2	Male	57	UPS	Chest wall	7.9	35	60	5	19
3	Female	39	Synovial sarcoma	Upper leg	8.2	30	60	10	13
4	Male	61	UPS	Lower leg	6.5	15	80	5	20
5	Female	71	UPS	Upper leg	7.0	20	20	60	23
6	Male	56	Myxofibrosarcoma	Lower back	17.5	15	50	35	9
7	Male	79	UPS	Buttock	10.2	35	45	20	13
8	Male	72	UPS	Buttock	12.7	20	50	30	20
9	Female	53	Spindle cell sarcoma, NOS	Chest	7.5	50	25	25	12
10	Male	54	Pleomorphic liposarcoma	Upper leg	21.1	90	10	0	8
11	Male	73	UPS	Upper arm	7.8	15	70	15	12
12	Male	37	Myxofibrosarcoma	Buttock	5.8	85	10	5	6
13	Male	44	Myxoid liposarcoma	Lower leg	10.6	5	0	50**	11
14	Female	38	Spindle cell sarcoma, NOS	Upper leg	10.8	5	70	25	16
15	Male	47	UPS	Upper leg	20.8	5	95	0	12

*Measured by a musculoskeletal radiologist on baseline anatomical MRI scan.

**This myxoid liposarcoma also showed 45% fatty maturation in the resection specimen.

Abbreviations: MRI = magnetic resonance imaging, %VC = percentage of viable tumor cells, %N = percentage of necrosis, %H/F = percentage of hyalinization or fibrosis, NOS = not otherwise specified, UPS = undifferentiated pleomorphic sarcoma.

Supplementary Figs. 1 and 2 show representative histograms of all qMRI parameters for a non-responder and responder, respectively.

The reasons for missing MRI data are listed in Supplementary Table 3.

### 3.2 Test-retest analysis

Repeatability metrics are reported in Table 2. Due to patients missing data for one or both of the baseline scans, repeatability metrics for ADC were based on 7 patients, for T_2_ and K^trans^ this was 13 patients. The Pearson correlation coefficient for individual subjects standard deviations versus means were −0.098 (p = 0.817), 0.760 (p = 0.003) and 0.626 (p = 0.022) for ADC, T_2_ and K^trans,^ respectively. Therefore, for ADC, the RC was used as threshold to indicate actual change, whereas for the other two qMRI parameters the %RC was used. The RC was 0.17 × 10^–3^ mm^2^/s for ADC and %RC was 5 % and 65 % for T2 and K^trans^, respectively.

**Table 2:** Repeatability metrics for ADC, T_2_ and K^trans^.

	**wSD (95 % CI)**	**RC**	**wCV (95 % CI)**	**%RC**
**ADC**	0.06 * 10^-3^ (0.03 *10^-3^, 0.09*10^-3^) mm^2^/s	0.17*10^-3^ mm^2^/s	5.8 % (3.7 – 7.3)	16 %
**T_2_**	4.8 (3.0, 6.7) ms	13.3 ms	2.0 % (1.6 – 2.2)	5 %
**K^trans^**	0.05 (0.03, 0.07) min^−1^	0.14 min^−1^	23.4 % (20.4 – 26.1)	65 %

Abbreviations: QIB = quantitative imaging biomarker, wSD = within-subject standard deviation, RC = repeatability coefficient, wCV = within-subject coefficient of variance, %RC = repeatability coefficient in percentages.

### 3.3 Changes in qMRI parameter values

**Fig. 3:**
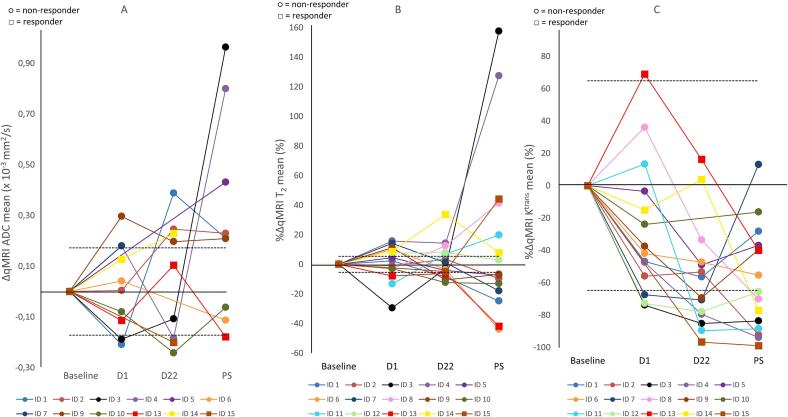
A: absolute change from B1 (ΔqMRI) for mean ADC, B: percentage change from B1 (%ΔqMRI) for mean T_2_; C: percentage change from B1 (%ΔqMRI) for mean K^trans^. The repeatability coefficient (RC) for ADC or repeatability coefficient in percentages (%RC) for T_2_ and K^trans^ derived from the test–retest analysis have been added as positive and negative value to the graphs as dotted lines. Histopathological responders have their data points as squares, non-responders have their data points as circles. Abbreviations: B1=first baseline scan, D1=day 1 scan, D22=day 22 scan, PS=pre-surgery scan.

Heterogeneous changes in all three qMRI parameter values during treatment can be observed both within and between patients (Fig. 3). At all timepoints, the majority of patients showed a decrease in mean K^trans^.

### 3.4 Correlation with histopathology

**Fig. 4:**
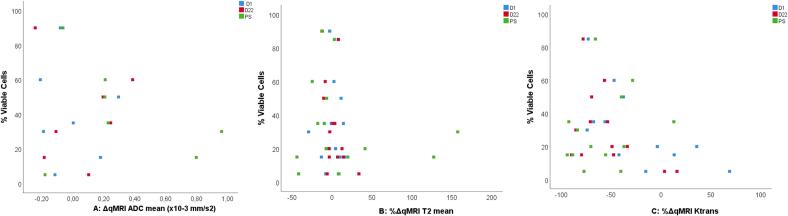
Scatter plots of A: ΔqMRI ADC mean at D1, D22 and PS versus % viable cells in the resection specimen, B: %ΔqMRI T_2_ mean at D1, D22 and PS versus % viable cells in the resection specimen, C: %ΔqMRI K^trans^ mean at D1, D22 and PS versus % viable cells in the resection specimen.

Fig. 4 shows scatter plots of ΔqMRI/%ΔqMRI of all three qMRI parameters at D1, D22, PS and the percentage of viable cells in the resection specimen.

Supplementary Table 4 shows the results of the association between ΔqMRI/%ΔqMRI of all three qMRI parameters and the percentage viable cells. No statistically significant association was found between percentage viable cells and ΔqMRI for ADC mean, between percentage viable cells (p = 0.561) and %ΔqMRI for K^trans^ (p = 0.710) and %ΔqMRI for T_2_ (p = 0.361).

Supplementary Fig. 3 shows correlation plots of ΔqMRI/%ΔqMRI of all three qMRI parameters and the percentage of necrosis and hyalinization/fibrosis. K^trans^ showed a significant statistical association with percentage necrosis (p = 0.017) and percentage hyalinization/fibrosis (p = 0.005) (Supplementary Table 5). T_2_ showed a statistically significant association with percentage necrosis (p = 0.018).

## 4 Discussion

This pilot study showed clear changes for fifteen STS patients in three qMRI parameters during and after treatment with concomitant radiotherapy and pazopanib, with heterogeneous changes in mean ADC and mean T_2_ and predominantly decreases in mean K^trans^. The next step would be to correlate qMRI parameter changes with response. In our study, we could not observe a clear association between percentage viable cells and qMRI parameter values, likely due to small sample size and heterogeneity of the tumors included. Although heterogeneous, the observed distinct changes in multiparametric imaging parameters could add relevant information to our understanding of the behavior of these tumors.

This study found a RC of 0.17 × 10^-3^ mm^2^/s for ADC (%RC of 16 %) and a %RC of 5 % for T_2_ and 65 % for K^trans^. To put this into perspective, the 2024 QIBA ADC profile proposes %RC values of 8 %, 27 %, 27 % and 15 % for brain, liver, prostate, breast lesions, respectively, and the 2023 QIBA K^trans^ consensus profile proposes a %RC value of 95 % for prostate tumors. However, it needs to be noted that QIBA requires a minimum of 35 patients for their statements and repeatability in our study was based on 7 patients for ADC and 13 patients for T_2_ and K^trans^. Other repeatability data in STS is scarce, with one other study reporting repeatability metrics [26]. However, as this study was in retroperitoneal sarcomas and the authors reported different repeatability metrics than we reported, comparison with our findings is hindered. The relatively high %RC for K^ktrans^ means only larger changes can be deemed as actual changes. If a more sensitive approach is warranted, repeatability of the K^ktrans^ technique needs to be improved. RC was used for ADC and %RC for T_2_ and K^trans^ following a correlation analysis between the variation and the mean of each parameter, which could influence future repeatability assessments for these parameters in STS.

In recent work by Kershaw et al. [27], changes in (semi-)quantitative imaging biomarkers in STS patients were assessed at two different time points, i.e. during RT treatment and post-treatment. During RT treatment, only T*_1_* values changed significantly, while ADC showed a significant change post-treatment. In another study by Xing et al. [7], maps derived from ADC and T_2_ were used to identify 5 distinct tumor habitats (i.e. high-T_2_ proteinaceous fluid, hypercellular tumor, necrosis, collagenous stroma, and fibrosis) to assess intra-tumoral heterogeneity in STS. In four out of seven patients, the hypercellular tumor habitats decreased in size between pretreatment and post treatment. The seven tumors mostly presented multiple habitats at all timepoints (before, during and after RT). The intratumoral heterogeneity in STS might be part of the explanation of the lack of correlation between qMRI parameters and viable cells found in this study, where we looked at the mean values across the whole tumor. Our data also suggests the presence of multiple habitats as in some cases multiple peaks were observed in the histograms of T2 and ADC at some time points (see supplementary Figs. 1 and 2). In future larger studies approaches such as tumor habitat imaging might be needed.

ADC is a promising biomarker for the evaluation of treatment response in STS, because of its inverse relation with the cellularity of tissues. Antitumor responses by radiotherapy and/or chemotherapy theoretically are associated with loss of viable tumor cell areas in STS, which should be reflected in rising ADC values, which was reported in the study by Kershaw et al [27]. Rising mean ADC in histopathological responders has been shown before in STS [8,28]. In our study we observed both increasing and decreasing ADC values during and after treatment, with no clear difference between responders and non-responders. Furthermore, no association between the change in ADC and the percentage of viable cells was found. All the patients with decreasing ADC at PS showed decreasing T_2_ as well. This concordance between ADC and T_2_ has been shown before in other tumor types [29]. However, of 6 patients with increasing ADC at PS, 4 patients showed decreasing T_2_. This is known from clinical experience in desmoid type fibromatosis tumors: in response to therapy, ADC increases and T_2_ decreases due to a decrease in cellularity and increase in collagen stroma (unpublished data, KvL). On the other hand, progressive disease is associated with increased T2 hyperintensity in the tumor [30,31].

All patients with significant changes in their K^trans^ values at *D22* and *PS* showed decreases. We expected larger decreases in K^trans^ in responders than in non-responders, as this has been shown in previous reports in STS [[9], [10], [11]]. This was not clearly discernible in our dataset, and might be explained by the small, heterogeneous sample size, however, difference in neo-adjuvant treatment might also be relevant. Furthermore, we included non-enhancing voxels (i.e. voxels with a K^trans^ value of 0) in our analysis. The general pattern we observed was an increase of the non-enhancing tumor areas over the course of the treatment. If non-enhancing voxels had been excluded, other time patterns might have resulted. To the best of our knowledge, we are the first to associate percentage necrosis and hyalinization/fibrosis with K^trans^ values, and percentage necrosis with T_2_ in STS. This is an interesting finding and could be explored in future tumor habitat studies.

The effects of the neo-adjuvant treatment on all three qMRI parameters seem to be time dependent; for all parameters the percentage of patients with a change larger than RC/%RC was higher at D22 and PS compared to D1.

One of the limitations of this study is the small sample size, this could have contributed to the heterogeneous findings and the repeatability results. This is especially relevant in the ADC RC, which was based only on 7 patients. More reports on qMRI parameter repeatability metrics in STS are needed to more accurately define change thresholds [13]. Missing patient data due to positioning errors contributed to this limitation. In the future we would recommend to use qMRI protocols with thicker slices, such that the whole tumor could be covered and positioning errors are prevented. Second, the study included different histological STS subtypes. Various subtypes react in different ways to treatment, prompting the question of whether it would be more sensible for future trials to evaluate multiparametric MRI parameters in cohorts of specific subtypes (or carefully selected categories of subtypes). Kousi et al [32] reported significant differences in the post-RT K^trans^ changes between responding and non-responding myxoid liposarcomas.

Third, ADC in our study has been calculated with different b-values than other studies [7,8], which could have led to less precise ADC results. The repeatability results we found, are for this protocol. Fourth, the presented linear mixed model does not capture complex temporal dynamics, as it assumes that any treatment effect captured by the pathological evaluation would need to already be present during image D1 and that changes over time in the qMRI parameter are not influenced by the pathology results.

To conclude, this pilot study reported relatively low repeatability coefficients for ADC and T_2_ and a higher repeatability coefficient for K^trans^. Furthermore, it showed clear heterogeneous changes in ADC and T_2_ values, along with predominantly decreasing K^trans^ values in fifteen STS patients. Although a clear correlation with percentage viable cells was not found, this study added valuable information on how to further implement these multiparametric MRI scans in treatment response evaluation in future STS clinical trials.

## Role of the funding source

Novartis funded this study, but their representatives had no role in the design nor in the conduct or analysis of the study.

## Declaration of competing interest

The authors declare that they have no known competing financial interests or personal relationships that could have appeared to influence the work reported in this paper.

## References

[1] van Houdt P.J., Saeed H., Thorwarth D., Fuller C.D., Hall W.A., McDonald B.A. (2021). Integration of quantitative imaging biomarkers in clinical trials for MR-guided radiotherapy: conceptual guidance for multicentre studies from the MR-Linac Consortium Imaging Biomarker Working Group. Eur J Cancer.

[2] Chen L., Liu M., Bao J., Xia Y., Zhang J., Zhang L. (2013). The correlation between apparent diffusion coefficient and tumor cellularity in patients: a meta-analysis. PLoS One.

[3] Tofts P.S., Brix G., Buckley D.L., Evelhoch J.L., Henderson E., Knopp M.V. (1999). Estimating kinetic parameters from dynamic contrast-enhanced T(1)-weighted MRI of a diffusable tracer: standardized quantities and symbols. J Magn Reson Imaging.

[4] Dekkers I.A., Lamb H.J. (2018). Clinical application and technical considerations of T(1) & T(2)(*) mapping in cardiac, liver, and renal imaging. Br J Radiol.

[5] Foltz W.D., Wu A., Chung P., Catton C., Bayley A., Milosevic M. (2013). Changes in apparent diffusion coefficient and T2 relaxation during radiotherapy for prostate cancer. J Magn Reson Imaging.

[6] van Schie M.A., van Houdt P.J., Ghobadi G., Pos F.J., Walraven I., de Boer H.C.J. (2019). Quantitative MRI changes during weekly ultra-hypofractionated prostate cancer radiotherapy with integrated boost. Front Oncol.

[7] Xing S., Freeman C.R., Jung S., Turcotte R., Levesque I.R. (2018). Probabilistic classification of tumour habitats in soft tissue sarcoma. NMR Biomed.

[8] Soldatos T., Ahlawat S., Montgomery E., Chalian M., Jacobs M.A., Fayad L.M. (2016). Multiparametric assessment of treatment response in high-grade soft-tissue sarcomas with anatomic and functional MR imaging sequences. Radiology.

[9] Huang W., Beckett B.R., Tudorica A., Meyer J.M., Afzal A., Chen Y. (2016). Evaluation of soft tissue sarcoma response to preoperative chemoradiotherapy using dynamic contrast-enhanced magnetic resonance imaging. Tomography.

[10] Meyer J.M., Perlewitz K.S., Hayden J.B., Doung Y.C., Hung A.Y., Vetto J.T. (2013). Phase I trial of preoperative chemoradiation plus sorafenib for high-risk extremity soft tissue sarcomas with dynamic contrast-enhanced MRI correlates. Clin Cancer Res.

[11] Xia W., Yan Z., Gao X. (2017). Volume fractions of DCE-MRI parameter as early predictor of histologic response in soft tissue sarcoma: a feasibility study. Eur. J. Radiol..

[12] Shukla-Dave A., Obuchowski N.A., Chenevert T.L., Jambawalikar S., Schwartz L.H., Malyarenko D. (2019). Quantitative imaging biomarkers alliance (QIBA) recommendations for improved precision of DWI and DCE-MRI derived biomarkers in multicenter oncology trials. J. Magn. Reson. Imaging.

[13] Boss M.A., Malyarenko D., Partridge S., Obuchowski N., Shukla-Dave A., Winfield J.M. (2024). The QIBA profile for diffusion-weighted MRI: apparent diffusion coefficient as a quantitative imaging biomarker. Radiology.

[14] van Meekeren M., Bovee J., van Coevorden F., van Houdt W., Schrage Y., Koenen A.M. (2021). A phase II study on the neo-adjuvant combination of pazopanib and radiotherapy in patients with high-risk, localized soft tissue sarcoma. Acta Oncol..

[15] Wardelmann E., Haas R.L., Bovée J.V., Terrier P., Lazar A., Messiou C. (2016). Evaluation of response after neoadjuvant treatment in soft tissue sarcomas; the European Organization for Research and Treatment of Cancer-Soft Tissue and Bone Sarcoma Group (EORTC-STBSG) recommendations for pathological examination and reporting. Eur. J. Cancer.

[16] Board WCoTE (2020).

[17] Le Bihan D. (2019). What can we see with IVIM MRI?. Neuroimage.

[18] Bos C.D.A., Sénégas J. (2009). Reference phantom validation of T2-mapping: maximum likelihood estimation of T2 from magnitude phased-array multi-echo data. Proc. Intl. Soc. Mag. Reson. Med..

[19] Schabel M.C., Parker D.L. (2008). Uncertainty and bias in contrast concentration measurements using spoiled gradient echo pulse sequences. Phys. Med. Biol..

[20] Murase K. (2004). Efficient method for calculating kinetic parameters using T1-weighted dynamic contrast-enhanced magnetic resonance imaging. Magn. Reson. Med..

[21] Parker G.J., Roberts C., Macdonald A., Buonaccorsi G.A., Cheung S., Buckley D.L. (2006). Experimentally-derived functional form for a population-averaged high-temporal-resolution arterial input function for dynamic contrast-enhanced MRI. Magn. Reson. Med..

[22] Bland J.M., Altman D.G. (1996). Measurement error. BMJ.

[23] Bates D., Mächler M., Bolker B., Walker S. (2015). Fitting linear mixed-effects models using lme4. J. Stat. Softw..

[24] Kuznetsova A., Brockhoff P.B., Christensen R.H.B. (2017). lmerTest package: tests in linear mixed effects models. J. Stat. Softw..

[25] Team RC (2021).

[26] Winfield J.M., Miah A.B., Strauss D., Thway K., Collins D.J., deSouza N.M. (2019). Utility of multi-parametric quantitative magnetic resonance imaging for characterization and radiotherapy response assessment in soft-tissue sarcomas and correlation with histopathology. Front. Oncol..

[27] Kershaw L., Forker L., Roberts D., Sanderson B., Shenjere P., Wylie J. (2021). Feasibility of a multiparametric MRI protocol for imaging biomarkers associated with neoadjuvant radiotherapy for soft tissue sarcoma. BJR|Open.

[28] Chodyla M., Demircioglu A., Schaarschmidt B.M., Bertram S., Bruckmann N.M., Haferkamp J. (2021). Evaluation of (18)F-FDG PET and DWI datasets for predicting therapy response of soft-tissue sarcomas under neoadjuvant isolated limb perfusion. J. Nucl. Med..

[29] Horger M., Vogel M.N., Beschorner R., Ernemann U., Wörner J., Fenchel M. (2012). T2 and DWI in pilocytic and pilomyxoid astrocytoma with pathologic correlation. Can. J. Neurol. Sci..

[30] Simonetti I., Bruno F., Fusco R., Cutolo C., Setola S.V., Patrone R. (2022). Multimodality imaging assessment of desmoid tumors: the great mime in the era of multidisciplinary teams. J. Pers. Med..

[31] Cassidy M.R., Lefkowitz R.A., Long N., Qin L.X., Kirane A., Sbaity E. (2020). Association of MRI T2 signal intensity with desmoid tumor progression during active observation: a retrospective cohort study. Ann. Surg..

[32] Kousi E., Messiou C., Miah A., Orton M., Haas R., Thway K. (2021). Descriptive analysis of MRI functional changes occurring during reduced dose radiotherapy for myxoid liposarcomas. Br. J. Radiol..

[33] Fuderer M., Wichtmann B., Crameri F., de Souza N.M., Baeßler B., Gulani V. (2025). Color-map recommendation for MR relaxometry maps. Magn. Reson. Med..

