# Supplementary material for: Multiparametric magnetic resonance imaging for radiotherapy response evaluation in high-risk soft tissue sarcoma: A pilot study
Source: Phys Imaging Radiat Oncol. 2025 Jul 25;35:100818. doi: 10.1016/j.phro.2025.100818 (PMC12347726; doi:10.1016/j.phro.2025.100818)
Supplement: Supplementary Data 1 [file mmc1.pdf]

8. Supplementary files

Supplementary table S1: Overview of scan parameters. For the quantitative maps two protocols were used, one for tumors on extremities and one for tumors in other areas. If two values are given (divided by /), the first value was for protocols for extremities

Parameter	Transverse T_1w+C	T_2 mapping	DWI	DCE***
**Sequence type**	Spin echo	Multi-echo spin echo	Echo planar imaging	3D spoiled gradient echo
**Field of view (mm^3)**	300x300x200*	300x300x75/ 250x520x75	300x300x75/250x400x75	260x297x75/262x449x75
**Acquired voxel size (mm^3)**	1x1x3	2x2x3	2.3x2.3x3	2.3x2.3x6
**Reconstructed voxel size (mm^3)**	0.60x0.60x3	1.20x1.20x3	1.25x1.25x3	1.30x1.30x3
**Parallel imaging factor**	2.5	4**	2	2.25/3
**Partial-Fourier factor**	no	0.61	0.62	no
**Repetition time (ms)**	450-750	2000-5000	4257	5
**First echo time +echo spacing (ms)**	10	32 + 16	65/68	1.9
**No. of echoes**	2	12	1	1
**Flip angle (degrees)**	90	90	90	20
**Fat suppression**	mDIXON	no	SPAIR	no
**b-values (s/mm2) (averages)**	-	-	0 (4), 50 (4), 600(8), 900(8)	-
**Diffusion directions**	-	-	3	-

**Dynamic scan time (s)**	-	-	-	4.9/5.0
**Receiver bandwidth (Hz)**	632.0/661.4	434.5	23.4/17.8	1992.8/1959.2
**Acquisition time (mm:ss)**	3:59/7:46	3:18/3:06	4:36/4:57	7:12

***** for the non-extremities protocol additional oversampling of 160 mm on both sides of FOV was used which prolonged the acquisition time

******a k-t undersampling scheme was used with a total acceleration factor of 4[34]

***Prior to the DCE sequence, a T1 mapping sequence using the variable flip angle approach was acquired for quantitative analysis of the DCE data. The protocol used the same sequence as DCE, but with flip angles of 3, 6, 10, 20 and 30 degrees, TR/TE was 20/4 ms.

Supplementary table S2: MR protocol details

A 32-channel posterior and anterior coil were used including a coil bridge to prevent deformation of the tumor due to the weight of the anterior coil.
For DCE measurements, 15 mL with an injection rate of 3 mL/s of 0.5 M Dotarem (Guerbet, Roissy CdG, France) was injected followed by a 30 mL saline flush using a power injector.
In order to achieve adequate temporal resolution in the DCE sequence, the field-of-view (FOV) was 75 mm in feet-head direction and therefore sometimes did not cover the entire tumor. The same FOV was used in the other qMRI sequences. When the FOV of the quantitative MRI sequences was too small to include the complete tumor, the MRI technician chose a representative central part of the tumor to be included in the scan. At later MR examinations, the technicians aimed to capture the same part of the tumor as included on previous scans.

Supplementary table S3: Reasons for missing MRI data

Case	qMRI parameter	Timepoint(s)	Reason for exclusion
5	ADC	B2, D1, D22	Incomplete fat suppression, resulting in artefact that overlaps with tumor
6	ADC	D22	Positioning error
7	ADC	D22, PS	Positioning error
7	ADC, T_2, K^{trans}	B1	Scans not according to protocol
8	ADC	All	Large deformations in DWI images, therefore the tumor could not be registered to the post-contrast T1 weighted scan.
10	ADC, T_2, K^{trans}	B1	Too much noise due to coil positioning
10	K^{trans}	D22	Severe movement of the patient during DCE acquisition
11	ADC	All	Positioning error
12	ADC	All	Large deformations in EPI, so tumor shape does not match anymore
14	ADC	B2, PS	Positioning error
15	ADC, T_2, K^{trans}	D1	Scan not according to protocol
15	ADC	PS	Positioning error

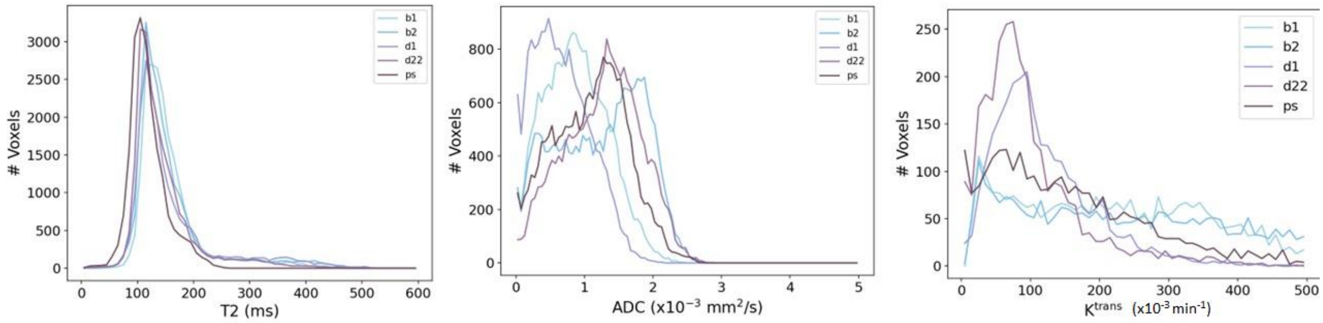

Supplementary figure S1: T_2map, ADC and K^{trans} histograms for ID 1 (non-responder)
Abbreviations: B1=first baseline scan, B2=second baseline scan, D1=day 1 scan, D22=day 22 scan, PS=pre-surgery scan

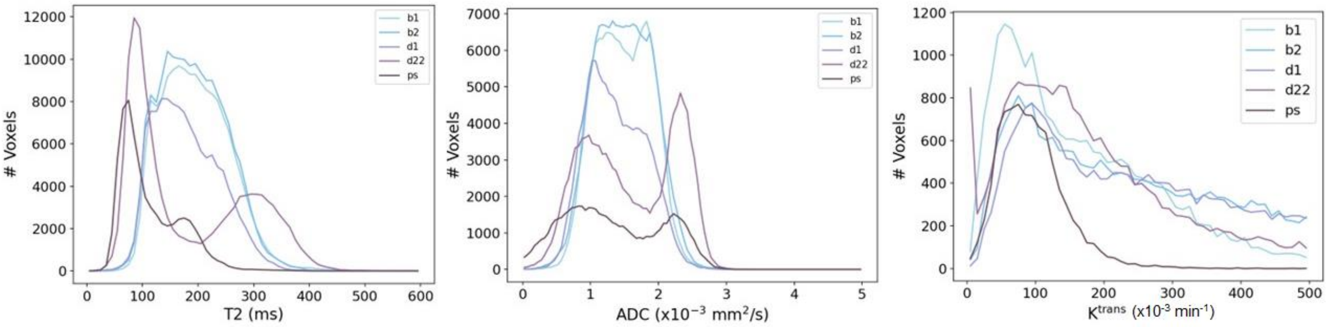

Supplementary figure S2: T_2map, ADC and K^{trans} histograms for ID 13 (responder)
Abbreviations: B1=first baseline scan, B2=second baseline scan, D1=day 1 scan, D22=day 22 scan, PS=pre-surgery scan

Supplementary table S4: Regression coefficient (B) along with the standard error (SE) for percentage of viable cells and time estimated from linear mixed models for three different outcomes: ΔqMRI ADC mean, %ΔqMRI K^{trans} mean, %ΔqMRI T_2 mean

**Model 1:** *ΔqMRI ADC mean (x10^{-3} mm^2/s)*			
	B (SE)	Std. Error (SE)	p-value
percentage of viable cells (%)	-0.001	0.002	0.561
Time (per time step)	0.126	0.065	0.064
**Model 2:** *%ΔqMRI K^{trans} mean (%)*			
	Estimate (B)	Std. Error (SE)	p-value
percentage of viable cells (%)	-0.105	0.276	0.710
Time (per time step)	-15.150	5.775	0.014*
**Model 3:** *%ΔqMRI T_2 mean (%)*			

	Estimate (B)	Std. Error (SE)	p-value
percentage of viable cells (%)	-0.182	0.198	0.361
Time (per time step)	7.147	6.480	0.276

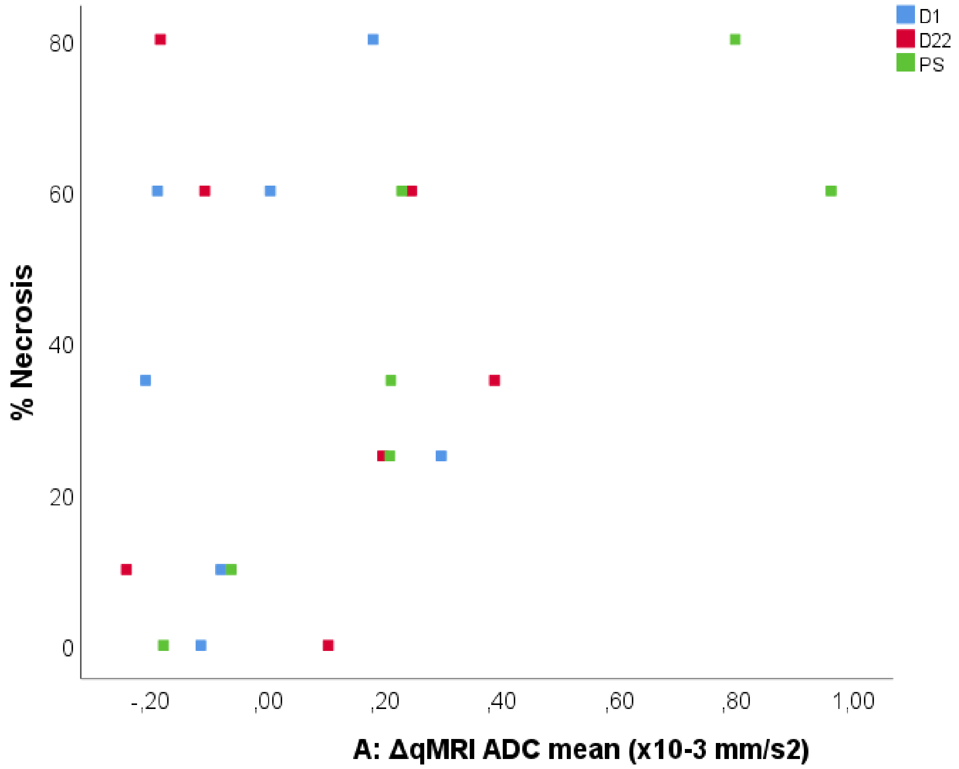

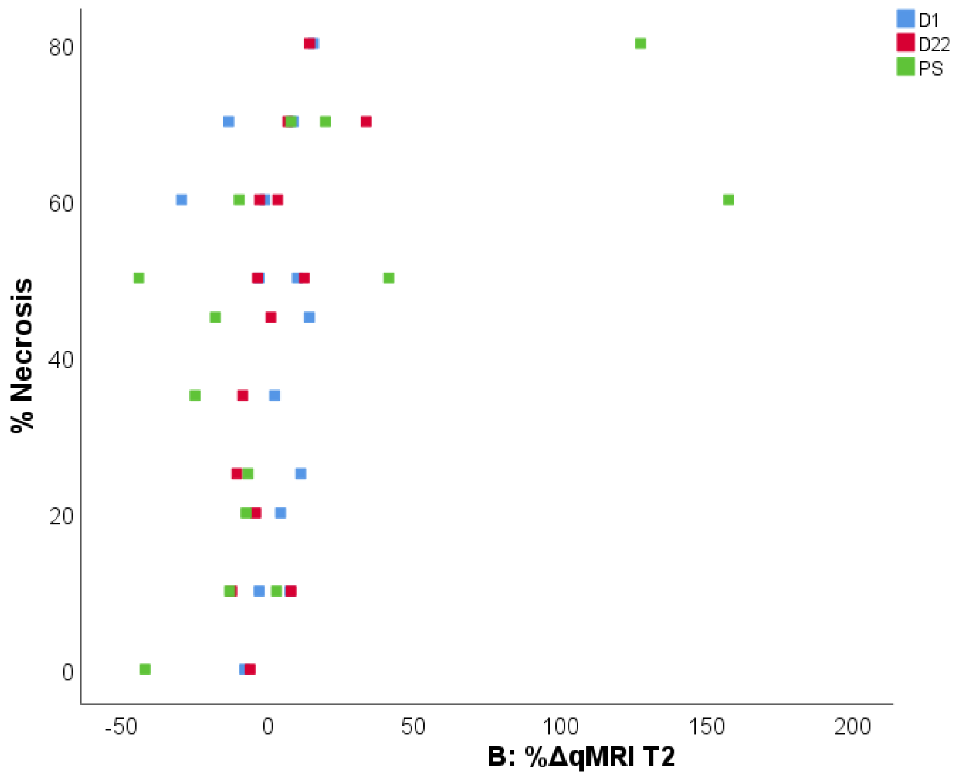

D1
D22
PS
% Necrosis
80
60
40
20
0
-50
0
50
100
150
200
B: %ΔqMRI T2

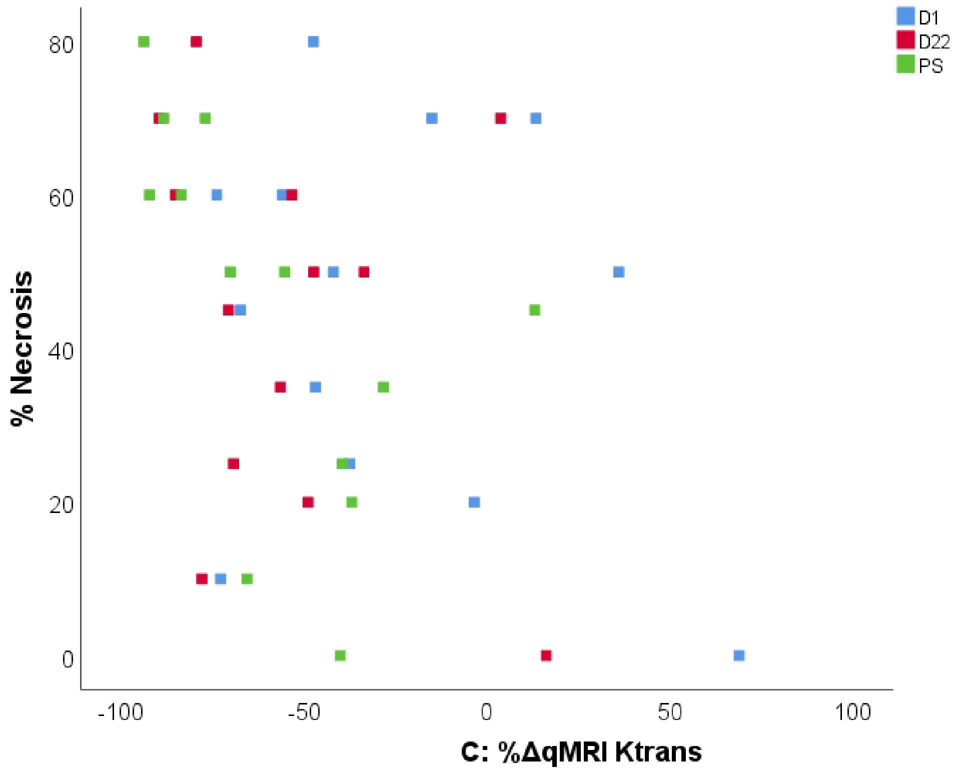

D1
D22
PS
% Necrosis
80
60
40
20
0
-100
-50
0
50
100
C: %ΔqMRI Ktrans

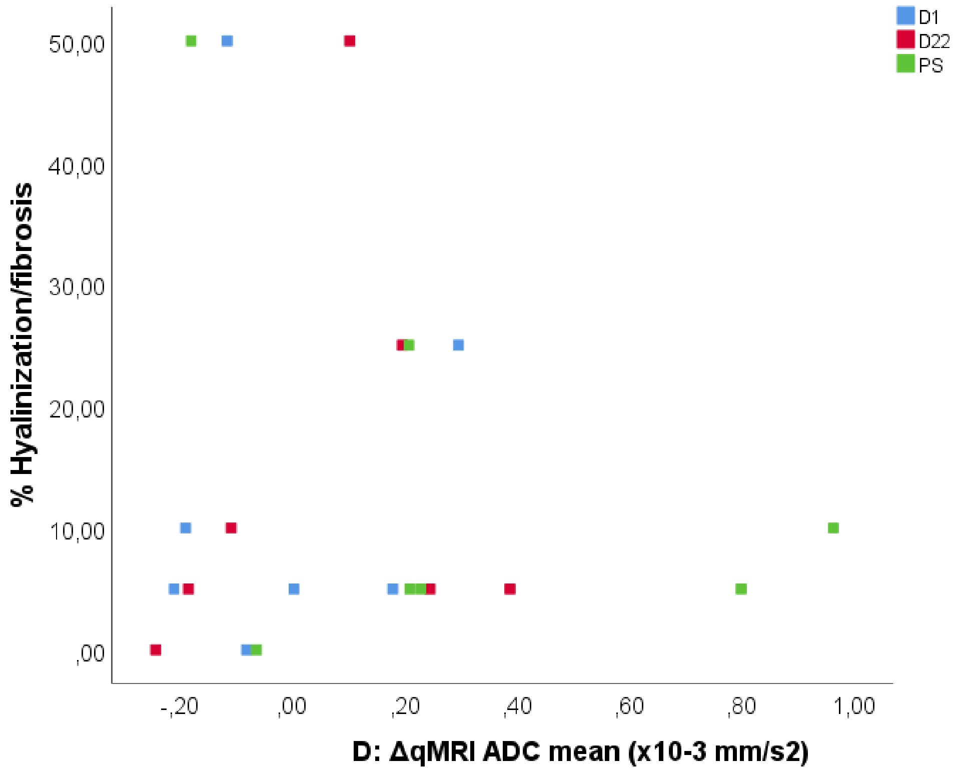
D1
D22
PS
50,00
40,00
30,00
20,00
10,00
,00
% Hyalinization/fibrosis
-,20
,00
,20
,40
,60
,80
1,00
D: ΔqMRI ADC mean (x10-3 mm/s2)

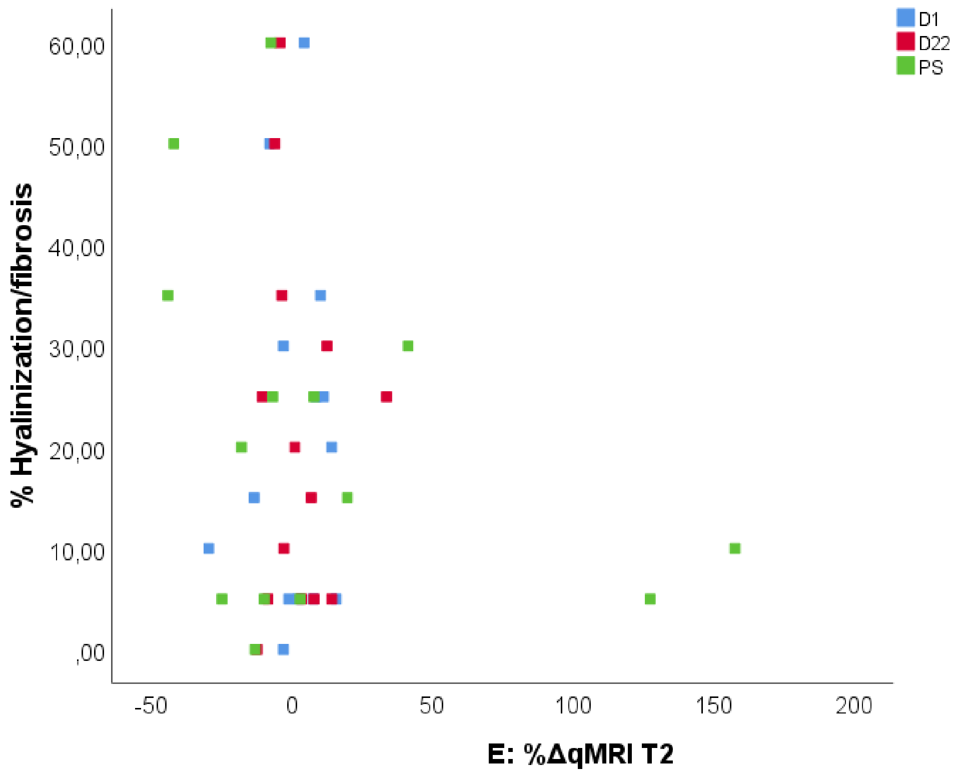
D1
D22
PS
60,00
50,00
40,00
30,00
20,00
10,00
,00
% Hyalinization/fibrosis
-50
0
50
100
150
200
E: %ΔqMRI T2

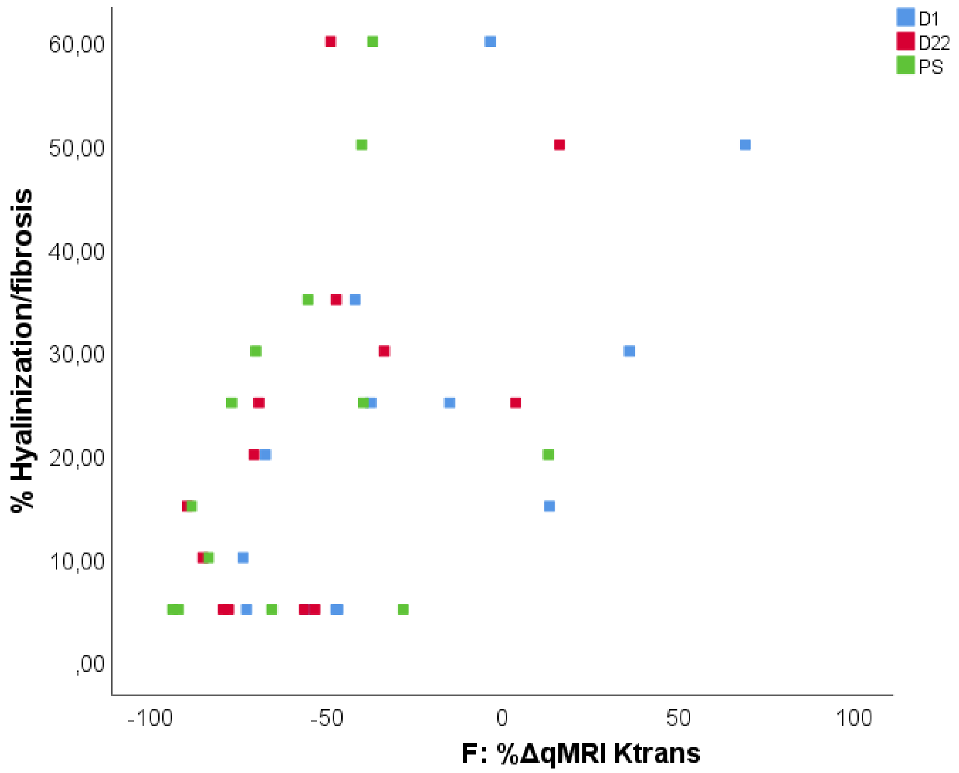

Supplementary Figure S3: Scatter plots of A: ΔqMRI ADC mean at D1, D22 and PS versus % necrosis in the resection specimen, B: %ΔqMRI T_2 mean at D1, D22 and PS versus % necrosis in the resection specimen, C: %ΔqMRI K^{trans} mean at D1, D22 and PS versus % necrosis in the resection specimen, D: ΔqMRI ADC mean at D1, D22 and PS versus % hyalinization/fibrosis in the resection specimen, E: %ΔqMRI T_2 mean at D1, D22 and PS versus % hyalinization/fibrosis in the resection specimen, F: %ΔqMRI K^{trans} mean at D1, D22 and PS versus % hyalinization/fibrosis in the resection specimen.

Supplementary Table S5: Regression coefficient (B) along with the standard error (SE) estimated from linear mixed models for percentage necrosis and percentage hyalinization/fibrosis for three different outcomes: Y1=ΔqMRI ADC mean, Y2=%ΔqMRI K^{trans} mean, Y3=%ΔqMRI T_2 mean

**Model 4:** *ΔqMRI ADC mean (x10^{-3} mm^2/s)*			
	Estimate (B)	Std. Error (SE)	p-value
percentage necrosis (%)	0.002	0.002	0.227
Time (per time step)	0.131	0.064	0.050
**Model 5:** *%ΔqMRI K^{trans} (%)*			
	Estimate (B)	Std. Error (SE)	p-value
percentage necrosis	-0.690	0.223	0.017*

(%)			
Time (per time step)	-14.644	5.700	0.016*
**Model 6:** *%ΔqMRI T_2 mean (%)*			
	Estimate (B)	Std. Error (SE)	p-value
percentage necrosis (%)	0.464	0.188	0.018*
Time (per time step)	6.510	6.114	0.293
**Model 7:** *ΔqMRI ADC mean (x10^{-3} mm^2/s)*			
	Estimate (B)	Std. Error (SE)	p-value
percentage hyalinization/fibrosis (%)	-0.000	0.003	0.955
Time (per time step)	0.125	0.065	0.068
**Model 8:** *%ΔqMRI K^{trans} (%)*			
	Estimate (B)	Std. Error (SE)	p-value
percentage hyalinization/fibrosis (%)	-1.008	0.293	0.005*
Time (per time step)	-15.034	5.827	0.016*
**Model 9:** *%ΔqMRI T_2 mean (%)*			
	Estimate (B)	Std. Error (SE)	p-value
percentage hyalinization/fibrosis (%)	-0.379	0.295	0.207
Time	7.065	6.420	0.278
